# Stigma and depression among heterosexual men and women living with HIV in the UK: Investigating a conceptual framework

**DOI:** 10.1371/journal.pone.0343610

**Published:** 2026-05-04

**Authors:** Ada R. Miltz, Janey Sewell, Fumiyo Nakagawa, Lorraine Sherr, Iain Reeves, Carole Kelly, Hannah Kitt, Valerie Delpech, Anu Fasanya, Alex Sparrowhawk, Vicky Lomas, Andrew Ustianowski, Vasiliki Papageorgiou, Colette J. Smith, Valentina Cambiano, Alison Rodger, Fiona C. Lampe

**Affiliations:** 1 Institute for Global Health, University College London, London, United Kingdom; 2 Homerton University Hospital NHS Foundation Trust, London, United Kingdom; 3 UK Health Security Agency, London, United Kingdom; 4 North Coast Population and Public Health, Northern New South Wales, Australia; 5 George House Trust, Manchester, United Kingdom; 6 UK-CAB, London, United Kingdom; 7 Regional Infection Unit, North Manchester General Hospital, Manchester, United Kingdom; 8 Royal Free London NHS Foundation Trust, London, United Kingdom; National Institute of Mental Health and Neurosciences: National Institute of Mental Health and Neuro Sciences, INDIA

## Abstract

**Background:**

Examining the relationship of HIV stigmatisation in different social settings with internalised HIV stigma, loneliness and depression among people with HIV, may help in further understanding HIV stigma and informing approaches and interventions to help to mitigate the impacts. The aim of this paper was to investigate among heterosexual men and women, a devised conceptual model of hypothesized causal connections between socio-demographic, HIV-related and psychosocial factors, measures of HIV stigmatisation, and depressive symptoms, in which feelings of loneliness because of HIV are theorised to mediate the relationship between internalised HIV stigma and depression.

**Methods and findings:**

This was investigated using structural equation modelling (SEM) and data from Positive Voices, a national cross-sectional study of people living with HIV in the UK (2022−2023). The analysis is based on participants who identified as heterosexual and were cis-gender (N = 1232). The mean age was 52 years; 48.5% were Black African, and 39.0% of white ethnicity. Twenty percent of participants were diagnosed with HIV within the last decade (2014−2022). Overall, 51.6% and 35.2% agreed or strongly agreed with the statements ‘I am ashamed of my HIV status’ and ‘I have poor self-esteem because of my HIV status’ respectively; 57.9% (n = 713/1232) agreed/strongly agreed with either item and were classified as positive for internalised HIV stigma. For the measure of having felt isolated/lonely because of HIV, the prevalence of responding with ‘yes, in the last year’ or ‘yes, but not in the last year’ was 14.2% and 11.7% respectively. The prevalence of depressive symptoms was 18.6% (PHQ-9 score ≥10). The data were consistent with the devised conceptual model. Of the factors investigated to be directly associated with internalised HIV stigma, the largest Beta coefficient was for the association with HIV stigmatisation in healthcare (Beta = 0.600; p < 0.001). The second largest was for having felt scared to be in public places because of HIV in the last year (Beta = 0.271; p < 0.001), closely followed by HIV stigmatisation from family (Beta = 0.269; p < 0.001). Of the factors investigated to be directly associated with depression, the largest Beta coefficient was for the association with having felt isolated/lonely because of HIV (Beta = 0.492; p < 0.001). The second largest was for the inverse association with resilience (Beta = −0.288; p < 0.001), followed closely by financial hardship (Beta = 0.267; p < 0.001), having ‘told most people in my life that I have HIV’ (Beta = 0.267; p < 0.001), and the inverse association with supportive network (Beta = −0.202; p < 0.001). The largest Beta coefficient for direct effects was for the association between internalised HIV stigma and having felt isolated/lonely because of HIV (Beta = 0.842; p < 0.001). Internalised HIV stigma was associated with depression indirectly through having felt isolated/lonely because of HIV (indirect Beta = 0.415; p < 0.001).

**Conclusions:**

Our findings add to existing literature by implicating a number of key factors that upon intervention may help prevent or attenuate symptoms of depression in people living with HIV, including reducing stigmatisation in healthcare, internalised HIV stigma, and loneliness/social isolation.

## Introduction

Although there have been profound advances in biomedical treatment that have transformed the lives of people living with HIV, HIV-related stigma remains a significant burden with implications for mental health [1–3]. Stigma can be understood as the social construction of a minoritised identity, leading to social oppression, discrimination, and exclusion [4–6]. Although stigma is an often resisted social process [4], the powerful nature of stigma as a symbolic apparatus that legitimizes inequalities of power that are based upon differential understandings of value and worth, means that the ability of oppressed and marginalised individuals to resist stigmatising forces can be limited [4]. Stigma, therefore, often affects the social interactions of those stigmatised and may lead to feelings of shame, a deeply personal sense of guilt, and social isolation. The dynamic of intersectional stigma, heightened discrimination based on combinations of gender, sexuality and ethnicity is increasingly acknowledged in HIV research [7]. The internalisation of stigmatising social attitudes often has consequences for the mental health and well-being of stigmatised populations [4,5]. In particular, stigma is thought to be a key factor that could explain the higher levels of depressive symptoms apparent among people living with HIV compared to the general population [8].

A recent meta-analysis of 17 cross-sectional studies among people living with HIV (2002–2019, eight from high-income countries) found that people who reported HIV-related stigma (as assessed by a variety of questionnaire tools that capture internalised and/or experienced stigma) were more likely to report depressive symptoms than those who did not (pooled odds ratio, OR 1.61 [95% CI: 1.38, 1.83]) [9]. Findings were similar in a previous meta-analysis of 24 studies among people living with HIV (1996–2013): the pooled correlation between HIV-related stigma and depressive symptoms was 0.403, p = 0.001 [10]. The association was also apparent across eight of the 24 studies that controlled for potential confounders (pooled correlation 0.301, p = 0.001 [10]). A systematic review of qualitative evidence (published 1996–2010) has shown that HIV-related stigma is a broad social phenomenon that manifests within multiple social domains, including within families, friendship groups, healthcare environments, and general society [1]. Separating these experiences of stigmatisation in different settings, and examining their relationship with internalised HIV stigma, and depression, may help in further understanding the effects of stigmatisation and informing approaches and interventions to help to mitigate the impacts.

One of the ways in which experiences or internalisation of stigma may lead to depression is through loneliness. Loneliness is defined as a distressing feeling that accompanies chronic perceived social isolation; the perception that one’s social needs are not being met by one’s social relationships [11]. Hawkley and Cacioppo (2010) state in their review of loneliness; “A perceived sense of social connectedness serves as a scaffold for the self – damage the scaffold and the rest of the self begins to crumble” [11]. Loneliness represents a critical concern for people living with HIV [12]. HIV-related stigma may lead to social isolation and loneliness; for instance, a person may restrict social interactions or isolate themselves in social situations because of HIV. In a systematic review of the literature on loneliness and social isolation among people living with HIV (2010–2024), the prevalence of loneliness ranged from 10%−58% across 15 studies, most of which were conducted in the U.S. [13] To date, three cross-sectional U.S. studies have provided some evidence, using structural equation modelling (SEM) methodology, that loneliness may be a mediator of the relationship between internalised HIV-related stigma and depression among people living with HIV [14–16].

The aim of this paper was to investigate among heterosexual men and women, hypothesized causal relationships between HIV stigmatisation in different social settings and internalised HIV stigma, and to assess the indirect effect of internalised HIV stigma on depression via feelings of loneliness due to HIV. This relationship was investigated among heterosexual men and women in the Positive Voices 2022 study, a national cross-sectional study of people living with HIV in the UK, using SEM and taking into account socio-demographic, HIV-related and psychosocial factors.

## Methods

### Positive voices study

‘Positive Voices 2022’ is the largest UK questionnaire study of people living with HIV [17]. In total, 4622 participants from 101 HIV outpatient clinics in England, Wales and Scotland, self-completed a questionnaire on demographic, socioeconomic, psychosocial, HIV-related and other health and lifestyle factors between April 2022 and March 2023. The current analysis is based on all Positive Voices 2022 participants who identified as straight or heterosexual and were cis-gender (gender identity is the same as sex assigned at birth) (N = 1564). The Positive Voices study was granted ethical approval by the London Harrow Research Ethics Committee (13/LO/0279) on March 28, 2013. The patient information leaflet provided information on the study and contact information for the Positive Voices team. Clinic staff were advised to tell each eligible participant that participation was voluntary and that their decision to participate would not affect their care. Consent was implied on voluntary completion of the questionnaire.

### Measures

#### Stigma.

In Positive Voices 2022, questions on HIV-related stigma were adapted from the Internalized AIDS-Related Stigma Scale [2] and from questions in a previous exploratory factor analysis of constructs of stigma with regards to sexual behaviour among gay, bisexual and other men who have sex with men (GBMSM) [18]. In the current study, seven constructs of stigmatisation were investigated, as shown below, with the survey questions used to define these constructs shown in brackets:

1. **Internalised HIV-related stigma** (“I am ashamed of my HIV status”; “I have poor self-esteem because of my HIV status”)
2. **HIV-related stigmatisation from family** (“Felt excluded from family activities”; “Felt that family members have made discriminatory remarks or gossiped about you”)
3. **HIV-related stigmatisation in healthcare** (“Felt that you were not treated well in a healthcare setting”; “Felt that you were refused healthcare or delayed treatment or a medical procedure”; “Heard healthcare staff gossiping about you (talking about you))”
4. **Anticipated HIV-related stigmatisation in healthcare** (“Felt afraid to go to healthcare services because someone may learn your HIV status”; “Avoided going to healthcare services when you needed to”; “Been worried that you would be treated differently to other patients by healthcare staff”)
5. **Rejected by friends because of HIV** (“Felt rejected by your friends”)
6. **Scared to be in public places because of HIV** (“Felt scared to be in public places”)
7. **Verbally harassed because of HIV** (“Been verbally harassed”)

For internalised HIV stigma (construct 1, above), participants were asked “How much do you agree with the following statements”, with response options: ‘strongly disagree’, ‘disagree’, ‘agree’, ‘strongly agree’, ‘prefer not to say’. For the remaining stigma constructs investigated (2–7), participants were asked “Because of your HIV status, have you ever experienced any of the following in your life?”, with response options; ‘no’, ‘yes in the last year’, ‘yes but not in the last year’, ‘don’t know’, ‘prefer not to say’.

In SEM, stigma constructs 1–4 were considered to be unobserved (latent) variables [19], with the observable survey questions shown in brackets above used to define each construct of stigmatisation using factor analysis, see ‘Statistical analysis’ section below.

For the two questions used to define internalised HIV stigma (construct 1), the response option ‘prefer not to say’ was treated as missing. If one of the two question responses was missing, this was imputed using the response to the other question. For the questions used to define constructs 2–4, response options were ordered as follows: ‘no’, ‘yes but not in the last year’, ‘yes in the last year’, in which the response option ‘don’t know’ was included in the ‘no’ category and the response option ‘prefer not to say’ was treated as missing. Where missing values were present for at least one but not all of the questions for a construct, missing responses were included in the ‘no’ category for that question. For constructs 5–7 (rejected by friends, scared and verbally harassed) each based on a single question, a binary variable was derived in each case; ‘yes in the last year’ or ‘no’, in which the latter category included ‘yes but not in the last year’, ‘don’t know’, ‘prefer not to say’ and missing responses.

When describing the correlates of stigma constructs in unadjusted modified Poisson regression analysis, a binary variable was defined in which participants were considered to have internalised HIV stigma if their response to at least one survey question in this construct was ‘agree’ or ‘strongly agree’; all other participants were included in the ‘no internalised stigma’ category (including responses ‘strongly disagree’, ‘disagree’, ‘prefer not to say’ and missing responses). For stigma constructs 2–4, stigmatisation was categorised as a three-level variable: (i) ‘no’ (‘no’, ‘don’t’ know’, ‘prefer not to say’ or missing) to all survey questions within each stigma construct), (ii) ‘yes but not in the last year’ (to at least one question, with the other response(s) being ‘no’, ‘don’t know’, ‘prefer not to say’, or missing), or (iii) ‘yes, in the last year’ (to at least one of the questions within each stigma construct). For stigma constructs 5–7, stigmatisation was considered to have occurred if the participant responded with ‘yes, in the last year’, and all other participants were included in the category ‘no’ (including responses ‘yes but not in the last year’, ‘don’t know’, ‘prefer not to say’ and missing responses).

### Loneliness due to HIV

Loneliness was measured using the question “Because of your HIV status, have you ever felt isolated/lonely?”. Response options included ‘no’, ‘yes in the last year’, ‘yes but not in the last year’, ‘don’t know’, ‘prefer not to say’. A binary variable was derived with categories; ‘yes in the last year’ or ‘no’, in which the latter category included ‘yes but not in the last year’, ‘don’t know’, ‘prefer not to say’ and missing responses.

### Depressive symptoms

Depressive symptoms were measured using the Patient Health Questionnaire (PHQ-9), a standardised inventory that enquires about frequency of occurrence of nine symptoms during the previous two weeks. Response options include: ‘not at all’ (coded as 0), ‘several days’ (coded as 1), ‘more than half the days’ (coded as 2), and ‘nearly every day’ (coded as 3) [20]. Missing values for individual questions within PHQ-9 were considered to indicate absence of that symptom; if all nine questions were missing the response was treated as missing. In SEM, depression was considered to be an unobserved (latent) variable, see ‘Statistical analysis’ section below, with the nine observable items on the PHQ-9 used to define the construct of depression. In unadjusted modified Poisson regression, a PHQ-9 total score of ≥10 was used to define depressive symptoms.

### Supportive network

Levels of a supportive network were measured using a modified version of the Duke-UNC Functional Social Support Questionnaire [21]. This scale enquired about the level at which participants received the following support from other people: ‘I have people who care what happens to me’, ‘I get love and affection’, ‘I get chances to talk to someone I trust about my personal or family problems’, ‘I get invitations to do things with other people’, ‘I get useful advice about important things in life’, and ‘I get help when I am sick in bed’. The response options (‘as much as I would like’, ‘almost as much as I would like’, ‘some but would like more’, ‘less than I would like’, or ‘much less than I would like’) were coded as 1–5, starting with ‘much less than I would like’ (coded as 1). Missing values for individual questions within this measure were imputed using the average value from the non-missing questions; if all six questions were missing the response was treated as missing. In SEM, supportive network was considered to be an unobserved (latent) variable, see ‘Statistical analysis’ section below, with the six observable items on the modified Duke-UNC Functional Social Support Questionnaire used to define the construct of a supportive network. In unadjusted modified Poisson regression, lacking a supportive network was measured using a cut-off total score of ≤10.

### Resilience

Resilience was measured using the Resilience Scale (RS) [22], which assesses an individual’s ability to cope with adversity and maintain well-being in difficult situations. RS has 14-items, each with a 7-point Likert scale (from ‘strongly disagree’to ‘strongly agree’). Scores are summed to create a resilience reading (range 14–98). Missing values for individual questions within this measure were imputed using the average value from the non-missing questions; if all 14 questions were missing the response was treated as missing. In SEM, resilience was considered to be an unobserved (latent) variable, see ‘Statistical analysis’ section below, with the 14 observable items on the RS used to define the construct of resilience. In unadjusted modified Poisson regression, a total score of 14–56 was considered to indicate “very low” resilience; 57–64 to indicate low resilience; 65–73 to indicate moderately low resilience; 74–81 to indicate moderate resilience; 82–90 to indicate moderately high resilience; and 91–98 to indicate high resilience [22].

### Other sociodemographic and HIV-related factors (Table 1)

Socio-demographic, HIV-related and psychosocial factors collected in Positive Voices 2022 that were identified as being relevant to depression and/or internalised HIV stigma among heterosexual men and women, based on the literature, were investigated in the SEM. These factors were used as either continuous variables or categorised as binary variables: *age* (as a continuous variable), *sex* (female or male), *ethnicity* (Black African or other/missing ethnicity), *financial hardship* based on the question “Do you have enough money to meet your basic needs (food, rent, gas, electricity, water etc)?” (yes [‘yes, always’/‘most of the time’/missing] or no [‘some of the time’/‘no’]), *currently have a main partner* (yes or no/missing), *religious beliefs are very important* based on the question “How important are religious beliefs to you?” (no [‘fairly’/‘not very’/‘not important at all’/‘not applicable’/missing] or yes [‘very important’]), *year of HIV diagnosis* (as a continuous variable), *told “most people in my life that I have HIV”* (no/missing or yes), and *strong endorsement of a statement about U = U* (undetectable = untransmissible) - “A person on HIV treatment with undetectable viral load cannot pass on HIV through sex” – (no [‘believe somewhat’/‘no, I don’t believe it’/‘not sure’/‘I don’t know what undetectable means’/missing] or yes [‘yes, strongly believe’]).

**Table 1:** Socio-demographic and HIV-related characteristics of heterosexual men and women living with HIV.

N = 1232 heterosexual cis-gender men (n = 498) and women (n = 734)		n (%)
Age (in years)	18-34	59 (4.8%)
	35-44	225 (18.3%)
	45-54	424 (34.4%)
	55-64	372 (30.2%)
	65+	152 (12.3%)
Ethnicity	White	481 (39.0%)
	Black African	597 (48.5%)
	Other Black ethnicity	47 (3.8%)
	Asian	41 (3.3%)
	Mixed ethnicity	41 (3.3%)
	Other or undisclosed	25 (2.0%)
Educational attainment	Primary or less	62 (5.0%)
	Upper secondary education	434 (35.2%)
	Technical & vocational	151 (12.3%)
	Undergraduate degree	280 (22.7%)
	Postgraduate degree	263 (21.3%)
	Other	19 (1.5%)
	Missing	23 (1.9%)
Employment status	Employed	830 (67.4%)
	Unemployed	100 (8.1%)
	Retired	136 (11.0%)
	Not working due to sickness or disabiliy	97 (7.9%)
	Student/carer/other	57 (4.6%)
	Missing	12 (1.0%)
Enough money to cover basic needs	Yes, always	481 (39.0%)
	Most of the time	373 (30.3%)
	Some of the time	246 (20.0%)
	No	118 (9.6%)
	Missing	14 (1.1%)
Currently have a main partner	No	480 (39.0%)
	Yes	713 (57.9%)
	Prefer not to say	25 (2.0%)
	Missing	14 (1.1%)
Religion	None	203 (16.5%)
	Spiritual, not religious	73 (5.9%)
	Christian	840 (68.2%)
	Buddhist	31 (2.5%)
	Hindu	4 (0.3%)
	Jewish	1 (0.1%)
	Muslim	37 (3.0%)
	Prefer not to say	16 (1.3%)
	Other	14 (1.1%)
	Missing	13 (1.1%)
Importance of religious beliefs	Very important	588 (47.7%)
	Fairly important	212 (17.2%)
	Not very important	128 (10.4%)
	Not important at all	60 (4.9%)
	Not applicable	203 (16.5%)
	Missing	41 (3.3%)
Year of HIV diagnosis	2019-2022	27 (2.2%)
	2014-2018	227 (18.4%)
	2008-2013	304 (24.7%)
	2002-2007	388 (31.5%)
	1996-2001	195 (15.8%)
	Pre 1996	91 (7.4%)

### Devising a conceptual model of causal connections between socio-demographic, HIV-related, psychosocial and stigmatisation factors, and depressive symptoms

**Fig 1:**
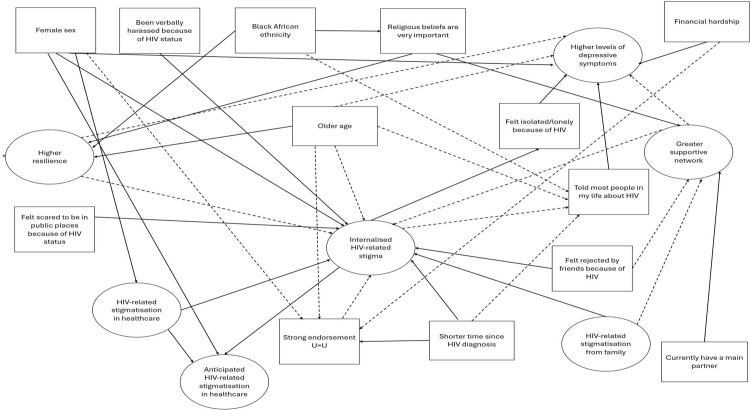
Overall conceptual model of hypothesised causal connections between socio-demographic, HIV-related and psychosocial factors, HIV-related stigmatisation, internalised stigma and depressive symptoms among heterosexual men and women living with HIV. Footnote: Inverse (negative) hypothesised relationships are indicated with a dotted line for the arrow.

A conceptual model of hypothesised causal relationships between all factors described above was devised (shown in Fig 1). This includes a hypothesised mechanism of causation; whereby feelings of isolation/loneliness because of HIV are theorised to mediate the relationship between internalised HIV stigma and depression. Hypothesized causal relationships were derived from previous findings of other research studies, although such relationships have not been proven to be causal. The postulated relationships are discussed in the context of the results of the conceptual model in the Discussion section.

### Statistical analysis

In this study, the main analysis used SEM. In SEM, the interpretative value of Beta coefficients extends only to that of comparing effect sizes to determine which factor is of ‘greater importance’ to the model. Therefore, to provide context, presented prior to the main SEM analysis, are unadjusted associations of socio-demographic, HIV-related, psychosocial and stigmatisation factors with: (i) internalised HIV stigma and (ii) depressive symptoms. χ^2^ tests and χ^2^ tests for trend were used to assess statistical significance and modified Poisson regression with a robust variance estimator was used to produce unadjusted prevalence ratios (PRs). These associations were not used to inform the conceptual model.

### Structural equation modelling (SEM) and confirmatory factor analysis

Although associations with internalised HIV stigma and depression are presented for all factors considered relevant in this study, some of these associations were not hypothesized to be causal and hence are excluded as potential pathways in the conceptual model. The set of hypothesized causal connections was then modelled within the framework of SEM to establish the consistency of the data with the devised conceptual model, which included a hypothesised mechanism of causation between internalised HIV stigma and depression, via loneliness/isolation.

Seven confirmatory factor analyses (CFA) were incorporated into the SEM model (for internalised HIV stigma, HIV stigmatisation from family, HIV stigmatisation in healthcare, anticipated HIV stigmatisation in healthcare, depressive symptoms, supportive network and resilience). In CFA, for each participant, a predefined latent variable is constructed from the average response to the observed survey items, which are weighted by the regression coefficients (factor loadings) for the relationship between the common variance among observed items and each of the observed items. In this study, treating depression, supportive network and resilience as latent variables instead of using existing scoring systems was considered appropriate in the context of SEM given that latent variables account for unreliability of measurement.

A mediator pathway was incorporated and assessed by specifying an indirect pathway in SEM. It was investigated whether internalised HIV stigma was associated with depression through feelings of isolation/loneliness because of HIV. A significant indirect effect indicates that a hypothesised intermediate factor may be on the causal pathway [23].

Analyses were performed using Stata and Mplus statistical software [24,25]. A generalised weighted least square based robust estimator (the mean and variance-adjusted WLS, WLSMV) was incorporated in the SEM due to the inclusion of binary/categorical outcome variables.

### Determining model fit

The Comparative Fit Index (CFI), the Tucker-Lewis Index (TLI), and the Root Mean Square Error of Approximation (RMSEA) test in SEM were used to guide the conceptual model fit. The model is considered to have a satisfactory fit if: CFI and TLI are ≥ 0.90 and RMSEA is ≤ 0.08. The model is considered to have a good fit if: CFI and TFI are ≥ 0.95 and RMSEA is ≤ 0.06 (higher 90% CI ≤ 0.08) [26,27]. Given the limitations of the χ^2^ test, this model fit index was not used [26].

### Analysis population

Participants were removed from all analyses if they left blank the entire questionnaire section for HIV-related stigma (n = 56 missing from 1564). Participants were also removed if they left blank both stigma survey questions used to define construct (1.) internalised HIV stigma (a further 120 participants were removed); if they left blank both questions used to define construct (2.) HIV stigmatisation from family (further 63 people); all three questions used to define construct (3.) HIV stigmatisation in healthcare (further 12 people); all three questions used to define construct (4.) anticipated HIV stigmatisation in healthcare (1 further person). This resulted in 252 exclusions. A further 58 people were excluded due to missing data on all items on one or more of the following questionnaire sections; depression (PHQ-9), resilience (RS), and/or supportive network (modified Duke-UNC). Finally, a further 22 participants were removed from the analysis as they had missing data on their year of HIV diagnosis. This resulted in 332 people being removed from the analysis, leaving 1232 participants included.

### Additional analyses

Two sensitivity analyses were conducted: (i) men who reported sex with at least one man (n = 13) and women who reported sex with at least one woman (n = 2) in the past three months were excluded from the SEM and (ii) participants with missing responses to socio-demographic factors (financial hardship (n = 14), currently have a main partner (n = 14), religious beliefs are very important (n = 41) and endorsement of U = U (n = 4)) were excluded from the SEM. Results are discussed in the text.

Three additional SEM analyses were conducted:

a) Two additional associations were included; between having told ‘most of the people in my life’ about HIV status and HIV stigmatisation from family and between having told ‘most of the people in my life’ about HIV status and rejection by friends because of HIV. The direct associations between having ‘told most people in my life that I have HIV’ and depression, and between internalised HIV stigma and having ‘told most people in my life that I have HIV’, were removed.
b) An additional direct association between internalised HIV stigma and depression was included.
c) Two additional associations were included; between being a Black African person and HIV stigmatisation in healthcare and between being a Black African person and anticipation of HIV stigma in healthcare. This was in order to address intersectional theory that intersecting stigmatised identities may lead to heightened experiences of HIV stigma [28].

Finally, it was investigated using χ^2^ tests whether the 332 participants who were removed from the analysis due to missing data (as described above) differed significantly from the 1232 participants included in the analysis, in terms of socio-demographic factors; age, sex, ethnicity, year of HIV diagnosis, financial hardship, religious beliefs are very important, having told most people about HIV, and endorsement of U = U.

## Results

### Descriptive statistics

Of the 4622 individuals who participated in the Positive Voices study, 1595 self-identified as straight/heterosexual, of whom, 1564 (98%) had the relevant gender information and reported being cis-gender (657 men and 907 women). After removing participants with missing values as described above (332 people in total), 1232 (498 men and 734 women) were included in this analysis.

The distribution of socio-demographic and HIV-related factors is presented in Table 1. In summary, the mean age was 52 years. Just under half of participants were Black African (48.5%), and 39.0% were of white ethnicity. Forty four percent of participants reported a university degree level of education, and 29.6% responded with ‘no’ or ‘some of the time’ to the question of having enough money to cover basic needs. Fifty-eight percent of participants reported a current main partner, and 47.7% reported that religious beliefs were ‘very important’ to them. About twenty percent of participants were diagnosed with HIV within the last decade (2014–2022). Fifty four percent strongly endorsed the statement about U = U.

The prevalence of stigmatisation among the heterosexual individuals included in this analysis is shown in Table 2, in which sub-headings indicate the construct of stigma that each question is considered to represent. Within the internalised HIV stigma construct, 51.6% and 35.2% agreed or strongly agreed with the statements ‘I am ashamed of my HIV status’ and ‘I have poor self-esteem because of my HIV status’ respectively; 57.9% (n = 713/1232) agreed/strongly agreed with either item and were classified as positive for internalised HIV stigma. For the measure of having felt isolated/lonely because of HIV, the percentages responding with ‘yes, in the last year’, ‘yes, but not in the last year’, or ‘no’ were 14.2%, 11.7%, and 67.0% respectively (3% of participants responded with ‘don’t know’, 1.7% with ‘prefer not to say’, and 2.4% had a missing response). The prevalence of depressive symptoms based a total PHQ-9 score across the nine questions of ≥10, was 18.6% (n = 229/1232).

**Table 2:** Prevalence of stigmatising processes among heterosexual men and women living with HIV.

N = 1232 heterosexual cis-gender men (n = 498) and women (n = 734)		n (%)
**Internalised HIV-related stigma**		
I am ashamed of my HIV status	Strongly agree	307 (24.9%)
	Agree	329 (26.7%)
	Disagree	275 (22.3%)
	Strongly disagree	228 (18.5%)
	Prefer not to say	88 (7.1%)
	Missing	5 (0.4%)
I have poor self-esteem because of my HIV status	Strongly agree	195 (15.8%)
	Agree	239 (19.4%)
	Disagree	398 (32.3%)
	Strongly disagree	361 (29.3%)
	Prefer not to say	26 (2.1%)
	Missing	13 (1.1%)
**HIV-related stigmatisation from family^a^**		
Felt excluded from family activities	Yes, in the last year	33 (2.7%)
	Yes, but not in the last year	68 (5.5%)
	No	1040 (84.4%)
	Don’t know	77 (6.3%)
	Prefer not to say	12 (1.0%)
	Missing	2 (0.2%)
Felt that family members have made discriminatory remarks or gossiped about you	Yes, in the last year	51 (4.1%)
	Yes, but not in the last year	115 (9.3%)
	No	930 (75.5%)
	Don’t know	119 (9.7%)
	Prefer not to say	10 (0.8%)
	Missing	7 (0.6%)
**HIV-related stigmatisation in healthcare^a^**		
Heard healthcare staff gossiping about you (talking about you)	Yes, in the last year	47 (3.8%)
	Yes, but not in the last year	89 (7.2%)
	No	1008 (81.8%)
	Don’t know	70 (5.7%)
	Prefer not to say	13 (1.1%)
	Missing	5 (0.4%)
Felt that you were not treated well in a healthcare setting	Yes, in the last year	65 (5.3%)
	Yes, but not in the last year	130 (10.6%)
	No	966 (78.4%)
	Don’t know	47 (3.8%)
	Prefer not to say	18 (1.5%)
	Missing	6 (0.5%)
Felt that you were refused healthcare or delayed treatment or a medical procedure	Yes, in the last year	45 (3.7%)
	Yes, but not in the last year	81 (6.6%)
	No	1041 (84.5%)
	Don’t know	48 (3.9%)
	Prefer not to say	5 (0.4%)
	Missing	12 (1.0%)
**Anticipated HIV-related stigmatisation in healthcare^a^**		
Felt afraid to go to healthcare services because someone may learn your HIV status	Yes, in the last year	168 (13.6%)
	Yes, but not in the last year	220 (17.9%)
	No	794 (64.4%)
	Don’t know	29 (2.4%)
	Prefer not to say	16 (1.3%)
	Missing	5 (0.4%)
Avoided going to healthcare services when you needed to	Yes, in the last year	98 (8.0%)
	Yes, but not in the last year	107 (8.7%)
	No	1001 (81.3%)
	Don’t know	15 (1.2%)
	Prefer not to say	6 (0.5%)
	Missing	5 (0.4%)
10. Been worried that you would be treated differently to other patients by healthcare staff	Yes, in the last year	161 (13.1%)
	Yes, but not in the last year	234 (19.0%)
	No	769 (62.4%)
	Don’t know	48 (3.9%)
	Prefer not to say	14 (1.1%)
	Missing	6 (0.5%)
**Felt scared to be in public places^a^**		
Felt scared to be in public places	Yes, in the last year	43 (3.5%)
	Yes, but not in the last year	51 (4.1%)
	No	1103 (89.5%)
	Don’t know	26 (2.1%)
	Prefer not to say	4 (0.3%)
	Missing	5 (0.4%)
**Been verbally harassed^a^**		
Been verbally harassed	Yes, in the last year	39 (3.2%)
	Yes, but not in the last year	63 (5.1%)
	No	1093 (88.7%)
	Don’t know	21 (1.7%)
	Prefer not to say	13 (1.1%)
	Missing	3 (0.2%)
**Felt rejected by friends because of HIV^a^**		
Felt rejected by your friends because of HIV	Yes, in the last year	38 (3.1%)
	Yes, but not in the last year	96 (7.8%)
	No	973 (79.0%)
	Don’t know	97 (7.9%)
	Prefer not to say	20 (1.6%)
	Missing	8 (0.6%)

^a^Participants were asked “Because of your HIV status, have you ever experienced any of the following in your life”.

### Unadjusted associations of socio-demographic, HIV-related, psychosocial and stigmatisation factors with internalised HIV-related stigma and depressive symptoms

Table 3 presents the unadjusted associations of factors with: (i) internalised HIV stigma and (ii) depressive symptoms.

**Table 3:** Unadjusted associations of socio-demographic, HIV-related, psychosocial and stigmatisation factors with internalised HIV-related stigma and depressive symptoms among heterosexual men and women living with HIV.

N = 1232 heterosexual cis-gender men and women^c^			Internalised HIV-related stigma (n = 713; 57.9%)^a^			Depressive symptoms (n = 229; 18.6%)^b^		
		N (%)	%	Unadjusted PR^d^ [95% CI]	Overall *p* value^d^	%	Unadjusted PR^d^ [95% CI]	Overall *p* value^d^
Sex	Male	498 (40.4%)	54.6%	1	0.060	16.3%	1	0.087
	Female	734 (59.6%)	60.1%	1.10 [1.00, 1.22]		20.2%	1.24 [0.97, 1.59]	
Age (in years)	18-34	59 (4.8%)	66.1%	1.17 [0.93, 1.47]	0.160	22.0%	1.34 [0.74, 2.44]	0.698
	35-44	225 (18.3%)	63.1%	1.12 [0.94, 1.32]		16.4%	1.00 [0.63, 1.59]	
	45-54	424 (34.4%)	54.3%	0.96 [0.81, 1.13]		18.6%	1.13 [0.75, 1.71]	
	55-64	372 (30.2%)	58.1%	1.03 [0.87, 1.21]		20.2%	1.23 [0.81, 1.85]	
	65+	152 (12.3%)	56.5%	1		16.5%	1	
Ethnicity	White	481 (39.0%)	61.5%	1	0.080	23.7%	1	<0.001
	Black Afr.	597 (48.5%)	54.8%	0.89 [0.80, 0.99]		13.6%	0.57 [0.44, 0.74]	
	Other^f^	154 (12.5%)	58.4%	0.95 [0.82, 1.10]		22.1%	0.93 [0.66, 1.31]	
Currently have a main partner	Yes	713 (57.9%)	56.4%	1	0.211	13.2%	1	<0.001
	No	519 (42.1%)	59.9%	1.06 [0.97, 1.17]		26.0%	1.97 [1.56, 2.50]	
Enough money to cover basic needs^e^	Yes^g^	868 (70.5%)	54.6%	1	<0.001	13.9%	1	<0.001
	No^h^	364 (29.6%)	65.7%	1.20 [1.09, 1.32]		29.7%	2.13 [1.69, 2.68]	
Lacking a supportive network^i^	No	1124 (91.2%)	56.9%	1	0.008	16.1%	1	<0.001
	Yes	108 (8.8%)	68.5%	1.21 [1.05, 1.38]		44.4%	2.76 [2.15, 3.54]	
Resilience^j^	High	378 (30.7%)	43.7%	1	<0.001	3.4%	1	<0.001
	Mod. High	268 (21.8%)	58.6%	1.34 [1.15, 156]		7.8%	2.28 [1.16, 4.47]	
	Mod.	181 (14.7%)	61.3%	1.40 [1.19, 1.65]		21.0%	6.10 [3.33, 11.17]	
	Mod. Low	142 (11.5%)	69.0%	1.58 [1.35, 1.85]		21.8%	6.35 [3.42, 11.78]	
	Low	99 (8.0%)	66.7%	1.53 [1.28, 1.83]		46.5%	13.51 [7.60, 24.00]	
	Very low	164 (13.3%)	70.7%	1.62 [1.39, 1.88]		48.8%	14.18 [8.13, 24.76]	
Religious beliefs are very important	No^k^	644 (52.3%)	60.7%	1	0.036	22.1%	1	0.001
	Yes	588 (47.7%)	54.8%	0.90 [0.82, 0.99]		14.8%	0.67 [0.53, 0.86]	
HIV-related stigmatisation from family	No^l^	1035 (84.0%)	55.7%	1	<0.001	15.5%	1	<0.001
	Yes, not last yr^m^	133 (10.8%)	69.9%	1.26 [1.11, 1.42]		31.6%	2.04 [1.53, 2.72]	
	Yes, last year^m^	64 (5.2%)	68.8%	1.24 [1.04, 1.47]		42.2%	2.73 [1.98, 3.76]	
HIV-related stigmatisation in healthcare	No^l^	951 (77.2%)	54.9%	1	<0.001	14.3%	1	<0.001
	Yes, not last yr^n^	181 (14.7%)	65.2%	1.19 [1.05, 1.34]		27.6%	1.93 [1.46, 2.56]	
	Yes, last year^n^	100 (8.1%)	73.0%	1.32 [1.16, 1.52]		43.0%	3.01 [2.29, 3.96]	
Anticipated HIV-related stigmatisation in healthcare	No^l^	680 (55.2%)	44.7%	1	<0.001	12.8%	1	<0.001
	Yes, not last yr^o^	295 (23.9%)	69.5%	1.55 [1.39, 1.74]		20.7%	1.62 [1.20, 2.18]	
	Yes, last year^o^	257 (20.9%)	79.4%	1.78 [1.60, 1.97]		31.5%	2.46 [1.89, 3.22]	
Felt scared to be in public places because of HIV (past year)	No^l^	1189 (96.5%)	56.9%	1	<0.001	16.9%	1	<0.001
	Yes^p^	43 (3.5%)	86.1%	1.51 [1.33, 1.72]		65.1%	3.85 [2.99, 4.96]	
Been verbally harassed because of HIV (past year)	No^l^	1193 (96.8%)	57.8%	1	0.623	17.8%	1	<0.001
	Yes^q^	39 (3.2%)	61.5%	1.07 [0.83, 1.37]		43.6%	2.45 [1.68, 3.58]	
Felt rejected by friends because of HIV (past year)	No^l^	1194 (96.9%)	57.3%	1	0.002	17.4%	1	<0.001
	Yes^r^	38 (3.1%)	76.3%	1.33 [1.11, 1.60]		55.2%	3.17 [2.32, 4.33]	
Felt isolated/ lonely because of HIV (past year)	No^l^	1057 (85.8%)	53.6%	1	<0.001	12.9%	1	<0.001
	Yes^s^	175 (14.2%)	84.0%	1.57 [1.44, 1.71]		53.1%	4.13 [3.35, 5.09]	
Told most of the people in my life that I have HIV	No	1106 (89.8%)	60.4%	1	<0.001	17.8%	1	0.032
	Yes	126 (10.2%)	35.7%	0.59 [0.47, 0.75]		25.4%	1.43 [1.03, 1.972]	
Time since HIV diagnosis	2020-2022	27 (2.2%)	77.8%	1.36 (1.04, 1.78)	0.008	25.9%	0.87 [0.43, 1.78]	0.061
	2014-2019	227 (18.4%)	63.9%	1.12 (0.91, 1.37)		17.2%	0.58 [0.38, 0.89]	
	2008-2013	304 (24.7%)	59.5%	1.04 (0.85, 1.27)		18.8%	0.63 [0.43, 0.94]	
	2002-2007	388 (31.5%)	54.1%	0.95 (0.78, 1.16)		16.8%	0.56 [0.38, 0.83]	
	1996-2001	195 (15.8%)	53.3%	0.93 (0.75, 1.16)		17.4%	0.59 [0.38, 0.91]	
	Pre 1996	91 (7.4%)	57.1%	1		29.7%	1	
Strong endorsement of U = U^t^	No^u^	565 (45.9%)	63.2%	1	<0.001	19.7%	1	0.380
	Yes^v^	667 (54.1%)	53.4%	0.84 [0.77, 0.93]		17.7%	0.90 [0.71, 1.14]	

^a^‘ Agree’ or ‘strongly agree’ to at least one question used to define stigma construct (1).

^b^PHQ-9 score ≥10.

^c^N = 498 men and N = 734 women.

^d^PR = prevalence ratio, p value by Wald test using modified Poisson regression with a robust variance estimator in order to produce unadjusted prevalence ratios.

^e^Food, rent, gas, electricity, water, etc.

^f^Including other Black ethnicities, Asian, mixed ethnicity, other ethnicities, and ‘Prefer not to say’ or non-response.

^g^‘Yes, always’, ‘Most of the time’ or missing.

^h^‘Some of the time’ or ‘No’.

^i^Based on a modified version of the Duke-UNC Functional Social Support Questionnaire. The total score across five questions for each participant was generated. A total score of ≤10 was considered to indicate a lacking supportive network, i.e., low levels of a supportive network.

^j^Based on the 14-item Resilience Scale (RS-14). The total score across 14 questions for each participant was generated. A total score of 14–56 was considered to indicate “very low” resilience; 57–64 to indicate low resilience; 65–73 to indicate moderately low resilience; 74–81 to indicate moderate resilience; 82–90 to indicate moderately high resilience; and 91–98 to indicate high resilience.

^k^‘Fairly’, ‘Not very’, ‘not important at all’, ‘Not applicable’ or missing.

^l^‘No’, ‘Don’t know’, ‘Prefer not to say’ or missing.

^m^The second category is; ‘Yes, but not in the last year’ to all questions used to define stigma construct (2), or ‘Yes, but not in the last year’ to one question and ‘No’ to the other. The third category is; ‘Yes, in the last year’ to at least one question used to define stigma construct (2).

^n^The second category is; ‘Yes, but not in the last year’ to all questions used to define stigma construct (3), or ‘Yes, but not in the last year’ to at least one question and ‘No’ to the other(s). The third category is; ‘Yes, in the last year’ to at least one question used to define stigma construct (3).

^o^The second category is; ‘Yes, but not in the last year’ to all questions used to define stigma construct (4), or ‘Yes, but not in the last year’ to at least one question and ‘No’ to the other(s). The third category is; ‘Yes, in the last year’ to at least one question used to define stigma construct (4).

^p^‘Yes, in the last year’ to stigma construct (6)

^q^‘Yes, in the last year’ to stigma construct (7)

^r^‘Yes, in the last year’ to stigma construct (5)

^s^‘Yes, in the last year’ to the question: “Because of your HIV status, have you ever felt isolated/lonely”.

^t^Based on belief in the statement: *“A person on HIV treatment with undetectable viral load cannot pass on HIV through sex”*

^u^‘Yes, believe somewhat’, ‘No, I don’t believe it’, ‘Not sure’, ‘I don’t know what undetectable means’, or non-response.

^v^‘Yes, strongly believe.’

The following socio-demographic, HIV-related and psychosocial factors were found to be associated with internalised HIV stigma; female sex (borderline association), white ethnicity compared to Black African, financial hardship, lacking a supportive network, lower levels of resilience (for very low vs. high resilience; PR 1.62 [1.39, 1.88]; p < 0.001), not stating that religious beliefs are very important, more recent HIV diagnosis, not telling ‘most people in my life that I have HIV’, and not indicating strong endorsement of U = U. HIV stigmatisation from family was associated with internalised HIV stigma, with the magnitude of association being similar for experiences in the last year and experiences prior to the last year; ‘yes, but not in the last year’ vs. ‘no’ PR 1.26 [1.11, 1.42] and ‘yes in the last year’ vs. ‘no’ PR 1.24 [1.04, 1.47]; p < 0.001. HIV stigmatisation in healthcare and anticipated HIV stigmatisation in healthcare were associated with internalised HIV stigma, with the magnitude of associations being stronger for experiences of stigma in the last year than prior experiences; ‘yes, but not in the last year’ vs. ‘no’ PR 1.19 [1.05, 1.34] and ‘yes in the last year’ vs. ‘no’ PR 1.32 [1.16, 1.52]; p < 0.001 for stigmatisation in healthcare; ‘yes, but not in the last year’ vs. ‘no’ PR 1.55 [1.39, 1.74] and ‘yes in the last year’ vs. ‘no’ PR 1.78 [1.60, 1.97]; p < 0.001) for anticipated stigmatisation in healthcare. Other measures of HIV stigmatisation were also associated with internalised stigma; having felt scared to be in public places (PR 1.51 [1.33, 1.72]; p < 0.001) and having felt rejected by friends (PR 1.33 [1.11, 1.60]; p = 0.002). Feeling isolated/lonely because of HIV was strongly associated with internalised HIV stigma (PR 1.57 [1.44, 1.71]; p < 0.001).

The following socio-demographic, HIV-related and psychosocial factors were found to be associated with depressive symptoms; white or other non-Black African ethnicity, not currently having a main partner, financial hardship, lacking a supportive network, lower levels of resilience (with a very strong association, for very low vs. high resilience; PR 14.18 [8.13, 24.76]; p < 0.001), not stating that religious beliefs are very important, telling ‘most people in my life that I have HIV’, and having been diagnosed with HIV pre 1996 as well as more recently (2020–2022) compared to 1996–2019. All measures of HIV stigmatisation were strongly associated with depressive symptoms; stigmatisation from family (‘yes, but not in the last year’ vs. ‘no’ PR 2.04 [1.53, 2.72], ‘yes in the last year’ vs. ‘no’ PR 2.73 [1.98, 3.76]; p < 0.001), stigmatisation in healthcare (‘yes, but not in the last year’ vs. ‘no’ PR 1.93 [1.46, 2.56], ‘yes in the last year’ vs. ‘no’ PR 3.01 [2.29, 3.96]; p < 0.001), anticipated HIV stigma in healthcare (‘yes, but not in the last year’ vs. ‘no’ PR 1.62 [1.20, 2.18], ‘yes in the last year’ vs. ‘no’ PR 2.46 [1.89, 3.22]; p < 0.001), having felt scared to be in public places (PR 3.85 [2.99, 4.96]; p < 0.001), been verbally harassed (PR 2.45 [1.68, 3.58]; p < 0.001), and having felt rejected by friends (PR 3.17 [2.32, 4.33]; p < 0.001). Feeling isolated/lonely because of HIV was strongly associated with depressive symptoms (PR 4.13 [3.35, 5.09]; p < 0.001).

The pattern of association of factors with depression was broadly similar to that for internalised stigma; the most notable difference was that the association between having told ‘most people in my life that I have HIV’ and lower levels of internalised stigma was reversed for depression, whereby telling most people was associated with greater depression. This complex relationship is discussed in the Discussion section. Finally, although resilience was associated with both depression and internalised stigma, the trend of increasing depressive symptoms with lower levels of resilience was striking – the prevalence of depression was almost 50% among people who reported very low levels of resilience, a 14-fold increase compared to the highest resilience category.

The factors investigated were hypothesized to be directly or indirectly associated with internalised HIV stigma, and/or depression in the conceptual model presented in Fig 1.

### Conceptual model of causal connections between socio-demographic, HIV-related, psychosocial and stigmatisation factors and depressive symptoms

Fig 1 presents the *a priori* hypotheses made about causal connections between socio-demographic, HIV-related, psychosoical and stigmatisation factors and depressive symptoms. Factors are labelled according to how they were modelled in the SEM, i.e., the label shown is the non-reference category for binary variables and the group furthest from the reference category for ordinal/continuous variables. Inverse (negative) hypothesised relationships are indicated with a dotted line for the arrow.

### Confirmatory factor analyses, SEM and path coefficients in conceptual model

**Fig 2:**
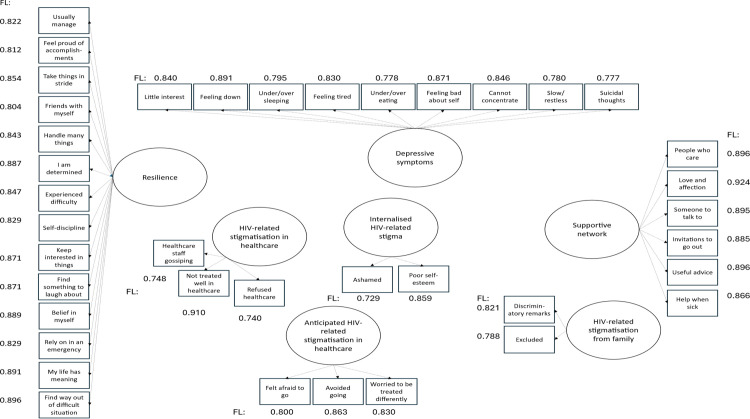
Factor loadings (FL) for observed items in confirmatory factor analyses among 1232 heterosexual cis-gender men and women living with HIV.

**Fig 3:**
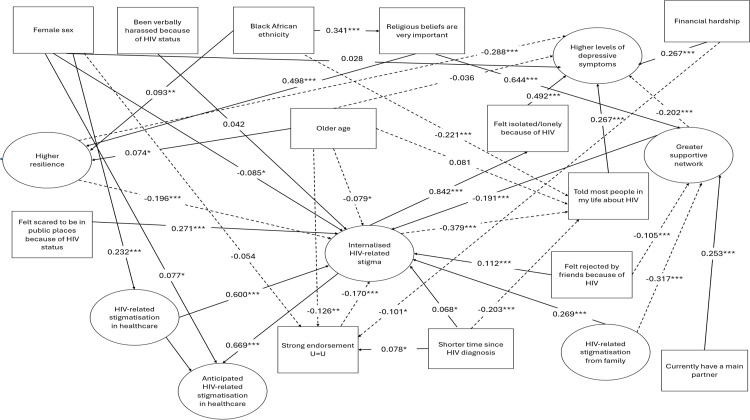
SEM of overall conceptual model of hypothesised causal connections between socio-demographic, HIV-related and psychosocial factors, HIV-related stigmatisation, internalised stigma and depressive symptoms among 1232 heterosexual cis-gender men and women living with HIV. Footnote: Model fit^a^: CFI: 0.962, TLI: 0.959, RMSEA: 0.048 (0.047, 0.050), Key: *p<0.05 **p<0.01 ***p<0.001, Solid line = positive association, Dotted line = negative (inverse) association.

Fig 2 presents the results of the confirmatory factor analyses (CFAs) for internalised HIV stigma, HIV stigmatisation from family, HIV stigmatisation in healthcare, anticipated HIV stigmatisation in healthcare, depressive symptoms, supportive network and resilience. Fig 3 presents the results of the SEM for the conceptual model (Fig 1), excluding factor loadings for each observed item. The Positive Voices data of 1232 cis-gender heterosexual men and women were considered to be consistent with the devised conceptual model, the fit indexes indicated a good model fit, see Fig 3. Most pathways specified were significant (p-values ≤0.05). Five pathways were not significant in SEM (p > 0.05), four of which were based on associations with sex or age, and one was between ‘verbally harassed because of HIV’ and internalised stigma. These pathways were not removed from the SEM since adjustment for sex and age was considered appropriate regardless of statistical significance, and as the number of participants reporting verbal harassment was limited (n = 39), power for this specific association may have been lower.

Of the factors investigated to be directly associated with internalised HIV stigma, the largest Beta coefficient was for the association with HIV stigmatisation in healthcare (Beta = 0.600; p < 0.001). The second largest was for having felt scared to be in public places because of HIV in the last year (Beta = 0.271; p < 0.001), closely followed HIV stigmatisation from family (Beta = 0.269; p < 0.001). Of the factors investigated to be directly associated with depression, the largest Beta coefficient was for the association with having felt isolated/lonely because of HIV (Beta = 0.492; p < 0.001). The second largest was for the inverse association with resilience (Beta = −0.288; p < 0.001), followed closely by financial hardship (Beta = 0.267; p < 0.001), ‘told most people in my life that I have HIV’ (Beta = 0.267; p < 0.001), and the inverse association with supportive network (Beta = −0.202; p < 0.001). The largest Beta coefficient for direct effects was for the association between internalised HIV stigma and having felt isolated/lonely because of HIV (Beta = 0.842; p < 0.001).

In terms of the indirect pathway specified, internalised HIV stigma was associated with depression indirectly through having felt isolated/lonely because of HIV (indirect Beta = 0.415; p < 0.001).

### Additional analyses

In sensitivity analysis (i), the findings in SEM were very similar when excluding men who reported sex with men (n = 13) and women who reported sex with women in the past three months (n = 2). In sensitivity analysis (ii), the findings in SEM were very similar when excluding participants with missing responses to socio-demographic factors.

In additional SEM analysis a), having told ‘most people in my life’ about HIV status was inversely associated with HIV stigmatisation from family (Beta = −0.507; p < 0.001) and inversely associated with rejection by friends because of HIV (Beta = −0.204; p < 0.001). The model coefficients remained very similar for all other pathways in SEM. In additional analysis b), when including an additional direct link between internalised HIV stigma and depression, the p-value for the direct effect was 0.075 (Beta = 0.201). In additional analysis c), being Black African was inversely associated with HIV stigmatisation in healthcare (Beta = −0.180; p=<0.001), and there was no evidence for a statistically significant association with anticipating HIV stigmatisation in healthcare (Beta = 0.010; p = 0.783).

The additional analysis investigating differences between the 332 participants who were removed from the analysis due to missing data and the 1232 included in the analysis found modest associations for a number of factors. Participants were more likely to be removed from this analysis due to missing data if they were; older (13.2%, 14.8%, 20.9%, 25.5%, 22.8%; for age groups 18–34, 35–44, 45–54, 55–64, and 65 + respectively; p = 0.005), men compared to women (24.1% vs. 19.2%; p = 0.019), of (non-African) Black ethnicity (34.7% vs. those of White, Black African, Asian, mixed, and other ethnicity, 14.3%, 24.5%, 25.5%, 18.0% respectively; p < 0.001), had financial hardship (23.2% vs. 18.0%; p = 0.018), and did not strongly endorse the statement about U = U (21.9% vs. 17.7% for strongly agree; p = 0.039).

## Discussion

### Summary of main findings

The Positive Voices data of 1232 cis-gender heterosexual men and women were considered to be consistent with the devised conceptual model of causal connections between socio-demographic, HIV-related and psychosocial factors, stigmatisation measures and depression, presented in this paper. Internalised HIV stigma was associated with depression indirectly through having felt isolated/lonely because of HIV. Based on a comparison of Beta coefficients in SEM, it appears that exposure to stigmatisation, particularly experiencing stigma in healthcare settings, and experiencing stigma from family, may play an important role in internalisation of stigmatising attitudes. For depression, the factors found to be of greatest importance in the model were having felt isolated/lonely because of HIV status and lower levels of resilience. The strongest association in the conceptual model for direct effects was for the association between internalised HIV stigma and having felt isolated/lonely because of HIV.

### Prevalence of and factors associated with stigma among people living with HIV

In the current study of cis-gender heterosexual men and women, just over half of participants (57.9%) were considered to have internalised HIV stigma, defined by agreement or strong agreement with the statement(s) ‘I am ashamed’ and/or ‘I have poor self-esteem’ because of my HIV status. In a recent review, the prevalence of HIV stigma (including internalised stigma) among people living with HIV was also high across 40 included studies (published up to April 2023), with a range of 19% (95% CI: 18.9%, 20.1%) to 88% (95% CI: 85%, 91%) [29]. Comparison across studies is difficult due to the use of multiple composite measures to assess different manifestations of stigma (internalised, experienced and/or anticipated), across different settings and communities. In the current study, the prevalence of having felt/experienced stigmatisation in the past year was: 5.2% (an additional 10.8% not in the last year) from family and 8.1% (an additional 14.7% not in the last year) in healthcare. The prevalence of having felt scared to be in public places, been verbally harassed, and rejected by friends because of HIV, in the past year, was 3.5%, 3.2%, and 3.1% respectively. The prevalence of anticipating stigmatisation in healthcare in the past year was higher at 20.9% (an additional 23.9% not in the last year). These findings suggest that although recent experiences of stigmatisation may be somewhat uncommon, expectations/fear of being stigmatised and internalisation of stigmatising attitudes remains relatively common for heterosexual men and women living with HIV.

In this SEM analysis, older age, female sex, greater number of years since HIV diagnosis, strong endorsement of U = U, greater supportive network, and higher levels of resilience were found to be associated with lower levels of internalised stigma among this sample of heterosexual men and women. These findings are in line with those in the above-described review [29], whereby older age and greater number of years living with HIV was significantly associated with an overall decreased level of stigma, but with a small effect size: pooled Beta coefficient for age across 16 studies; −0.04 [95% CI: −0.28, −0.01] and pooled Beta coefficient for years living with HIV across six studies; −0.17 [95% CI:-0.31, −0.03]. Higher levels of social support were associated with lower levels of stigma (pooled Beta coefficient across 12 studies; −0.34 [95% CI: −0.52, −0.16]). However, being a woman was associated with higher levels of stigma (pooled Beta coefficient across 11 studies; 0.47 [95% CI: 0.11, 0.84]) compared to men. In the SEM in the current study, women were somewhat less likely than men to have internalised stigma but more likely than men to report HIV stigmatisation in healthcare and anticipation of stigma in healthcare. It is possible that sexism, and dynamics of power and hierarchy between patient and clinician, may make women more susceptible to HIV-related stigma in a healthcare setting [30]. These findings may be consistent with intersectional theory that HIV and female gender lead to heightened experiences of stigma [28]. However, being Black African was not associated with greater HIV stigmatisation in healthcare or anticipation of HIV stigmatisation in healthcare in the SEM, see additional analysis c) in Results. Possibly, greater levels of resilience and the role of religion in providing supportive community may mitigate the stresses of racism.

In the current study, reporting that religious beliefs are ‘very important’ was strongly associated with a greater supportive network and higher levels of resilience. There is some evidence that community elements of religious traditions may potentially play a role in circumventing the impact of stigma on the lives of people living with HIV [31–33], offering a pathway out of the cycle of shame and isolation that may follow an HIV diagnosis [34,35]. This is in line with findings from a recent systematic review of studies (published 2000–2024) investigating the association of spirituality with health outcomes among people with a serious condition (“terminal, late-stage or catastrophic illness, or during end-of-life or palliative care”) [36]. Of all the measures of spirituality (e.g., frequency of prayer, spiritual salience), a prominent theme was the consistent positive association of more frequent religious/spiritual service attendance (vs. less or no attendance) with favourable health outcomes, including reduced depression incidence (33% reduction in odds in longitudinal meta-analysis [37,38]). Religious services might include themes of acceptance and forgiveness that may provide comfort and alleviate self-condemnation for some people. Religious community involvement may also offer a sense of belonging and social support as part of a welcoming and supportive community [32,33]. However, religion intersects with power and identity. Although community elements of religious traditions and faith may offer hope, meaning and purpose to some people living with HIV, some may feel concerned that their HIV status, sexuality and/or gender identity may not be acceptable to their religious institution, which may be stressful and isolating, causing tension in balancing identity with faith [39].

In this study of heterosexual men and women, overall 68% reported their religion as Christian, 3.0% as Muslim, 3% as Buddhist, 0.3% as Hindu, and 0.1% as Jewish (17% reported no religion and 6% reported being spiritual but not religious).Black African people were more likely to agree with the statement that ‘religious beliefs are very important to me’. It has been consistently documented in the U.S. that ethnic minority members report higher religiosity/spirituality [32,40]. In a 2015 U.S. longitudinal study of 122 people living with HIV (aged 36–65 years), at baseline, Black African Americans were more likely to report greater daily spirituality and more engagement in private religious practices (as measured by the Brief Multidimensional Measure of Religiosity, BMMRS), after adjusting for social support, resilience, ‘grit’, life satisfaction, and duration of HIV diagnosis [32]. Religious institutions often serve as important community structures and social resources for ethnic minority group members, who may be seeking culturally appropriate and language friendly faith-based experiences and social support and services [32,40]. In the current study, Black African people were more likely to have higher levels of resilience. The experience of living with minority stress, may lead to greater opportunities for community participation and connectedness, and ability to cope with adversity [32,41,42].

A comparison of effect sizes in this SEM study provides further insight into which factors may be of greatest importance to internalised stigma among this sample of heterosexual men and women. Findings suggest that the experience of discrimination and rejection from those who might be expected to be helping or supporting you, in healthcare settings, and in the case of family, loving you unconditionally, may play a vital role in determining one’s sense of self-worth and appraisal of what it means to be living with HIV.

### Loneliness as a mediator of the association between internalised stigma and depression among people living with HIV

In the SEM in this study, Internalised HIV stigma was associated with depression indirectly through having felt isolated/lonely because of HIV (indirect Beta = 0.415; p < 0.001), in line with previous studies. This was after accounting for age, sex, financial hardship, resilience, supportive network, and sharing of HIV status.

In a systematic review of the literature on loneliness and social isolation among people living with HIV (2010–2024, majority of studies from U.S.), 17 studies investigated the relationship between loneliness and depression. With the exception of two studies conducted in India, loneliness/isolation was found to be associated with higher likelihood of depressive symptoms [13]. A number of other studies have investigated relationships between stigma, loneliness and depression using SEM methodology. In a U.S. cross-sectional study (2016–2017) of 146 older people living with HIV (≥50 years old, 86% African American), loneliness mediated the association between internalised stigma (on the internalized AIDS-Related Stigma Scale) and depressive symptoms (CESD-R) [14] in SEM that accounted for sociodemographic factors (indirect effect = 0.16; p < 0.001). This was similar to results in the current study. In another U.S. cross-sectional study (2013–2014) of 1168 women currently on ART (2013–2014), internalised HIV-related stigma (negative self-image subscale of the revised HIV Stigma Scale) was found to have an indirect and negative effect on ART adherence mediated via loneliness (R-UCLA Loneliness Scale) and depression (CES-D) [15]. In a U.S. online cross-sectional survey of people living with HIV (N = 181, published 2018), there was a significant indirect effect of internalised stigma (negative self-image subscale of the HIV Stigma Scale) on poor sleep quality via higher levels of loneliness (UCLA Loneliness Scale) and more depressive symptoms [16]. In a context other than HIV, the mediating role of loneliness in the relationship between stigma and depression has also been found in a cross-sectional study of people recruited from mental health-care facilities in Poland, who met the diagnosis of ‘non-affective psychotic disorder’ (n = 110, published 2014). The authors reported that loneliness (De Jong Gierveld Loneliness Scale) fully mediated the association between internalised stigma (Internalised Stigma of Mental Illness scale) and depression (Calgary Depression Scale for Schizophrenia) [43].

Further insight into this relationship comes from a qualitative study of nine focus group discussions with 37 people living with HIV in England [42]. Participants shared how ongoing navigation of social misconceptions and ignorance led to being selective and careful about sharing their HIV status, when and with whom, and so feeling unable to be ‘one’s true self’ in social settings. Living in this ‘restricted’ way and having to hide part of oneself in some areas and spaces of life may make connections with others difficult and reinforce and intensify feelings of isolation and loneliness, with consequences for one’s mental health.

This study includes measures of positive factors such as resilience that may affect relationships and serve as a protective factor against internalised stigma and depression. Findings in this study indicate the significant role that internalised stigma may play in feelings of loneliness for heterosexual men and women living with HIV, as observed in previous qualitative work [42]. This was the strongest association in the SEM, with a large effect size (Beta = 0.842; p < 0.001 in SEM, PR 1.57 [1.44, 1.71]; p < 0.001 in unadjusted analysis).

### Other factors associated with depression among people living with HIV

The prevalence of depressive symptoms (PHQ-9 ≥ 10) among this sample of heterosexual men and women was 18.6%. Financial hardship, lower levels of resilience, lacking a supportive network, telling most people about HIV, and loneliness because of HIV was found to be associated with greater depression in unadjusted modified Poisson regression and SEM, which is in line with previous findings in the UK [44–48].

In the current study of heterosexual men and women, opposite associations of having told ‘most of the people in my life’ about HIV with depression and internalised HIV stigma were found – telling most people was associated with greater depression and lower levels of internalised stigma. In additional SEM analysis a), having told most people about HIV was associated with less HIV stigmatisation from family (Beta = −0.507; p < 0.001) and less rejection by friends because of HIV (Beta = −0.204; p < 0.001). This suggests that sharing one’s HIV status with most people may be more likely if there are supportive family and friends, which protects against internalised stigma, and that negative experiences may occur with wider disclosure in other contexts, such as with work colleagues, neighbours or other acquaintances. An association between high levels of social disclosure of HIV-status and depressive symptoms was also found among MSM in a previous UK study [47]. There may also be other mechanisms by which having told most people about HIV is linked to symptoms of depression, and/or unmeasured confounding factors in this association, such as personality type and coping style.

The SEM findings further emphasize the significance of loneliness for mental health among heterosexual men and women living with HIV. Even after accounting for financial hardship and level of supportive network, established risk factors for depression in the general population, one’s perceived social isolation as a result of HIV was independently and strongly associated with greater depressive symptoms.

### Strengths and limitations of this study

Given the cross-sectional design of the Positive Voices study, it is not possible to be assured of the direction of associations. The hypothesized causal sequence assessed in SEM may operate in the other direction, such that depression leads to feelings of loneliness/isolation, which in turns leads to higher levels of internalised stigma. However, there is some evidence in support of loneliness as a causal factor of depression in the general population – in a longitudinal study using a nationally representative cohort of adults aged 50 years and older in England, loneliness at baseline was associated with greater depression severity during a 12-year follow-up period [49]. Furthermore, recent analyses of five consecutive annual assessments of loneliness and depressive symptoms have shown that loneliness predicts increases in depressive symptoms over one year intervals, but depressive symptoms do not predict increases in loneliness over those same intervals [50]. Therefore, there is support for the hypothesised direction of association in the current study.

We included only cis-gender heterosexual individuals in this analysis. Due to small numbers of trans and non-binary individuals, it was not possible to include these individuals and stratify the sample to investigate the conceptual model separately for different gender identities. Trans and gender diverse people may also have different experiences of stigmatisation, and in different settings, which would require consideration of an appropriately tailored conceptual model. Similarly, considering stigma among gay and bisexual people (and people of other sexual minoritised identities) requires a suitably tailored conceptual model [51,52].

It is possible in the current study that the prevalence of loneliness is underestimated, as participants were asked whether they ‘felt lonely/isolated because of HIV’, which may not capture people who feel lonely but do not consider their loneliness to be as a result of living with HIV. More general data from, for instance, using the UCLA Loneliness Scale, was not collected. However, our measure of loneliness allowed the specific hypothesis about loneliness due to HIV to be tested.

In the Positive Voices survey, participants were asked if they had ‘experienced’ stigmatisation, with many items worded as ‘felt’ stigma, i.e., ‘felt excluded’, ‘felt rejected’ ‘felt that you were refused’ etc. As a result, participants may have experienced that form of stigmatisation and/or they may have perceived but not experienced it. It was possible in this study to separate felt/experienced stigmatisation in healthcare from anticipated (future expectations of) stigmatisation in healthcare, however this was not possible in other settings (such as stigmatisation from family or friends). In addition, the derived constructs of stigmatisation investigated in this paper were not validated scales.

Our results may also be affected by study non-response and missing data. For example, participants excluded from this analysis due to missing data (n = 332), were more likely than those included (n = 1232), to be older and of Black ethnicity (factors that tended to be associated with lower internalised stigma), but also more likely to report financial hardship and not strongly endorse the statement about U = U (factors associated with higher stigma). Therefore, the prevalence of different forms of stigma may be slightly overestimated or underestimated among our analysis population. However, interrelationships between different factors and HIV stigmatisation may be expected to be less affected by these exclusions.

Finally, there may be unmeasured confounding in this study, such as the nature of family relationships and attachments, and experiences of trauma (e.g., intergenerational), abuse, neglect and/or other forms of adverse childhood experiences, which may affect the degree of internalisation of negative attitudes, loneliness, and symptoms of depression.

### Implications for intervention

In a recent systematic review and meta-analysis of 67 randomised controlled trials (RCTs) of psychosocial interventions for adults living with HIV in high-income countries (published 2008−2023) [53], only seven included HIV-related stigma as an outcome. The meta-analysis demonstrated a small intervention effect on stigma, based on seven studies, which was not statistically significant (standardised mean difference, SMD −0.17 [95%CI: −0.35, 0.02]). Only three of the seven studies focused on stigma as the primary endpoint, two were for women with HIV, and one for individuals newly entering HIV care. Interventions were provision of an iPod touch with a video about the experiences of being a women living with HIV created from a qualitative metasynthesis of women’s lived experiences [54], face-to-face counselling drawing on motivational interviewing principles to facilitate adjustment to living with HIV [55], and a peer support workshop to foster social support and reduce stigma for African American women with HIV [56]. Findings in this current study provide support in favour of designing interventions to address stigmatization that may be experienced in a healthcare environment, and possibly from family members, in order to prevent and reverse the internalization of stigmatizing attitudes about HIV and its impact on one’s sense of self-worth and social relations. This is likely to require interventions targeted at healthcare staff as well as fostering skills and self-efficacy needed for effective healthcare seeking and management for people living with HIV. Alongside this, community elements of religious traditions may play a role in circumventing the impact of stigma on the lives of people living with HIV [34,57]. It has been suggested that it may be appropriate for healthcare providers ask about spirituality and faith to identify the influences of religion/spirituality on the health and well-being of their patients, and to help shape patient-centred care [36].

In the literature review on loneliness among people living with HIV (2010–2024) [13], it was suggested that social prescribing, which stresses the importance of training that familiarises practitioners with available resources at local community levels, may better meet the holistic needs of patients and potentially be of benefit for people experiencing loneliness. In the review of RCTs of psychosocial interventions described above [53], none of the 67 eligible interventions explicitly included social prescribing which remains a clear evidence gap. Psychosocial interventions that aim to address loneliness in people living with HIV, through for instance, social prescribing, may have the potential to particularly impact on depression.

## Conclusion

Findings in this study suggest that HIV stigmatisation in healthcare and HIV stigmatisation from family play a key role in the internalisation HIV stigmatising attitudes. Interventions that aim to reduce stigmatisation in healthcare, internalised HIV stigma, and loneliness/social isolation may help to prevent or attenuate symptoms of depression in heterosexual men and women living with HIV.
